# A Large Social Experiment

**Published:** 1918-05-04

**Authors:** 


### THE INSTITUTIONAL LIBRARY.

### A LARGE SOCIAL EXPERIMENT.

In our issue of March 16 we drew attention to the report of a committee of scientific men on the physiological action of alcohol, and took note of the limited amount of confident knowledge which actually exists on this subject. The recognition of this limitation is the first step towards its removal, and the report in question is merely the preliminary stage of an investigation which, we hope, will replace ignorance by ascertained and unquestioned facts. This will be a gain to science and a contribution to one aspect of a subject which has been prolific in controversies. But the pharmacological action of alcohol on the human body, important as such a study undoubtedly is, by no means exhausts the questions which find in the habitual use of alcohol a common centre. Whatever a scientific committee may say on this topic will hardly produce a striking or prompt influence on a custom which is deeply rooted in the national habits, and which, in some fashion or other, meets what is felt by many people to be a human need. Whether such convictions are, or are not, challenged by science, they exist as large facts of experience, and, sad though it may seem to the investigator, experience and habit and custom are apt to prevail even against doctrines endorsed by the sanction of laboratory demonstration. The alcohol question in its social and political relations cannot therefore expect to be seriously modified by findings which, whatever be their scientific merit and impartial quality, will leave untouched many larger and wider issues. The war has raised these issues in a very acute form. It has done more than this. In the restrictions which have been compelled by the war there has been included an immense experiment in which have been tested such questions as the influence of the alcoholic habit upon the efficiency and endurance of the workers and the ability of legislative action to modify this habit. Such questions in the past have given rise to endless discussions, but in recent years they have been put to the actual test of experience, and on a scale so large as to yield results which can hardly be challenged. The experiment has been made, not under artificial but under natural conditions, and upon men and women engaged in ordinary circumstances in working for their livelihood. Into this life and atmosphere has come the Central Control Board (Liquor Traffic) with its restrictive regulations, and the results of these regulations are for all to see. Here, then, is no mere academic debate or scientific inquiry detached from actuality or limited to some narrow issue. On the contrary, the facts are gathered from the observation of large masses of people living and working—but for one condition—under normal circumstances, and this one condition is a limitation of the opportunities of taking alcoholic drink.

The effects of such an experiment are, of course, of national importance. Whatever other disputes have existed in reference to alcohol, there has been universal agreement that the habits of our people in this respect leave much to be desired; that alcohol taken in excess is harmful, both to the individual and to the community; and that there is urgent need for reform if the health and efficiency of the nation are to be improved. How this reform could be accomplished was a question which raised the most acute controversies, and partly for this, and partly for other reasons, prudent politicians were disposed to refrain from legislative enterprises. Further, there was a widespread conviction that such enterprises promised little success, and it was a commonplace of the debate that people could not be made sober by Act of Parliament. There were, it is true, heroic souls who controverted this proposition, and who, true to their principles, would fain have abolished the drink traffic without further word said. But these were merely voices in the wilderness, and most persons with practical minds regarded them as hindrances rather than helpers in the cause of reform. Thus it came about that one of the most thorny of social questions seemed beyond the capacity of the Legislature, and not a few counsellors advised us to trust to the gradual extension of education and to social improvement—factors which, truth to tell, could boast some measure of triumph.

But, as we have already pointed out, an experiment which, but for the war, would certainly have been impossible, has actually been in operation, and everyone interested in his country's welfare will be anxious to learn what teachings have resulted from the trial. The desired information is now at the disposal of the public in a book which has been prepared by Mr. Henry Carter, and to which a preface has been contributed by Lord d'Abernon.* Here are described in detail the regulations issued by the Board of Control and the effects of these regulations on the habits and lives of the people of this country. The question discussed, it will be understood, is not the popular debate between total abstinence and moderate drinking, nor is it a lamentation over the evils of drunkenness. What the Board of Control had to face was the relation of the drink traffic as it actually existed to national efficiency, and to decide how far such a motive demanded the intervention of official control. It was an early lesson of the war that time was wasted and work inefficiently performed as a result of excessive drinking by a certain proportion of the workers. From this conclusion issues the responsibility of the Board, and it is here that is to be found the motive for all their regulations. These regulations have lessened the hours of sale of alcoholic drinks, have raised their price and diminished their strength, and, on

* *The Control of the Drink Trade.* By Henry Carter. London: Longmans, Green and Co. 1918. Pp. 323 + xvi. Price 7s. 6d. net.

**THE INSTITUTIONAL LIBRARY—(*continued*).**

the positive side, have secured opportunities for the obtaining of food and non-alcoholic beverages by munition and other workers. The effects in detail may be found in Mr. Carter's book. Stated broadly, they show a decrease in excessive drinking which is quite without parallel in our history. Whether such restrictions can be or ought to be maintained in time of peace is a matter for the country to decide. But the results most certainly dispose of the argument that legislation has no ability over the social aspects of the drink traffic. In face of them it is difficult to believe that Parliament can be allowed to proclaim itself helpless in regard to a question which is of vital importance to the nation, and on which it is now proved official control can exercise a large and salutary influence.

**The Edinburgh School of Surgery before Lister.** By ALEXANDER MILES, Surgeon to the Royal Infirmary, Edinburgh. 8vo. Eight Illustrations. Pp. vi + 220. (London: A. and C. Black, Ltd. 1918. 5s. net.)

IF a small, this is yet a well-written and worthy contribution to the history of surgery. It appeals more to the Edinburgh man than to the Scot at large, and more to the Scot than to the Englishman, but it is by no means devoid of interest for the latter; for though it recounts primarily the history of surgery in Edinburgh, with excellent, if brief, biographies of Edinburgh surgeons, yet the School of Surgery in Edinburgh has always been important, and representative of the teaching of surgery in the world at large. Even before Lister's day, many important improvements in surgery emanated from Edinburgh, and, moreover, several of the most distinguished of London surgeons, including Liston and Fergusson, were trained in Edinburgh, and owed to the reputation they had made there the invitations that brought them to posts in London hospitals.

It is very difficult for the surgeon of to-day to realise what surgery was before Lister's day, and still more difficult for him to imagine what surgery was before the introduction of anæsthetics. Equipped with these two stupendous advantages, surrounded with a multitude of instruments and appliances nicely adapted to every one of his requirements, the student of to-day is, as a rule, utterly ignorant of the steps by which the present perfection of technique has been attained, and he cannot grasp the importance of it all unless he is acquainted with the difficulties of his predecessors, and is able to follow their slow and laborious progress from primitive barber-surgery to present-day perfection. "Seeing only," says Mr. Miles, "the perfected results of the surgery of to-day, he fails, on the one hand, to realise the difficulties that had to be overcome by the pioneers of surgery, and, on the other, to grasp the real significance of the revolution effected by the master-mind of Joseph Lister."

The book is well written, well printed, and well illustrated, and is a worthy tribute to the Edinburgh School of Surgery, to the great men who contributed to make it, and to the history of surgery in general. The closing passage in the book may fittingly close this notice: "That the height of Lister's genius shone at its brightest in her midst is the lasting pride of the Edinburgh School; but 'many brave men lived before Agamemnon,' and, although we 'are without a divine poet to chronicle their deeds,' we would not willingly forget them."

**Manual of Splints and Appliances for the Treatment of Bone and Joint Injuries** (as supplied to the United States Army by the American Red Cross). (Published for the British Red Cross Society by Henry Frowde and Hodder and Stoughton. 1917. Oblong 12mo. Pp. xiv. and 206. Price 2s. 6d. net.)

IT is a friendly action on the part of the Medical Department of the United States Army and American Red Cross Society to allow the publication of this manual by the British Red Cross Society. The manual is the outcome of a desire to standardise the manufacture of splints and appliances used in military centres, and thus avoid some of the difficulties which occurred with our own wounded at the beginning of the war. The objects aimed at were (1) the possibility of quick manufacture and ease of distribution, (2) the comfort of the patient with avoidance of accessories, (3) universality of type and simplicity of construction. The manual is divided into four main sections. The first deals with detailed descriptions of splints and appliances for use at field and evacuation hospitals—*i.e.* for patients who must afterwards be moved. The second heading deals with splints and appliances recommended for use at base hospitals. The apparatus in these centres may necessarily be more elaborate, as the patient will not again be moved until his injuries have become consolidated. Part 3 treats of surgical dressings and accessory supplies, whilst the last section is devoted entirely to illustrations showing by means of outline diagrams the methods of applying the different forms of apparatus. Each set of illustrations is accompanied by letterpress indicating the uses to which the splint may be put, notes of the particular points to be observed in order to obtain the maximum of usefulness, and a reference to the detailed description of each splint as found in Parts 1 and 2. The drawings have been well rendered by Mrs. Cyrus W. Thomas, who appears to have given her services without fee or reward.

The use of splints and the application of bandages cannot be learnt from books, but with this manual in his hand everyone should be able to apply a splint with such ease that it performs its function and is comfortable to the wearer. The information is often elementary and the detail trivial, but successful surgery rests upon principles and detail. The value of the manual, therefore, is great, and its study is as necessary to the civil as to the military surgeon. Surgeon-General Sir George H. Makins contributes an introductory note, and the book is rounded off with a good index. The publishers have so arranged the size of the manual that it can be carried comfortably in a pocket of the military tunic.

**Blood Pictures: an Introduction to Clinical Hæmatology.** By CECIL PRICE-JONES, M.B.. (Bristol: John Wright and Sons, Ltd. 1917. Pp. 91 + iv. Price 6s. 6d. net.)

THIS is a strictly practical manual intended to encourage the practitioner to cultivate for himself a knowledge of clinical hæmatology. It gives working directions for the counting of the blood corpuscles, the estimation of hæmoglobin, and the preparation and interpretation of stained blood films. The author states what he himself has found of service, and avoids the discussion of alternative methods—a plan which, for the beginner, is the right method of instruction. He has the art of clear exposition, and his text is aided by several very successful coloured illustrations. Altogether he has produced a serviceable volume.

